# The composition of phenolic compounds in Chinese olive (*Canarium album* L.) cultivars and their contribution to the anti-inflammatory properties of the cultivars

**DOI:** 10.3389/fnut.2024.1334077

**Published:** 2024-02-19

**Authors:** Fangqing He, Yixuan Du, Zhuangguang Pan, Huize Zeng, Haolin Luo, Junyi Wang, Yuanming Sun, Meiying Li

**Affiliations:** ^1^Guangdong Provincial Key Lab of Food Safety and Quality, South China Agricultural University, Guangzhou, Guangdong, China; ^2^College of Food Science, South China Agricultural University, Guangzhou, Guangdong, China

**Keywords:** anti-inflammatory, antioxidant, *Canarium album* L., Chinese olive, phenolic compounds

## Abstract

**Objective:**

This study aimed to explore the phenolic compounds (PCs) present in three Chinese olive (*Canarium album* L.) cultivars and the contribution of these PCs to the anti-inflammatory activities of the cultivars.

**Methods:**

Ultra-high performance liquid chromatography coupled with hybrid quadrupole-orbitrap/mass spectrometry (UPLC-Q-Exactive/MS) was used to identify and quantify the PCs present in three Chinese olive cultivars, “Na zhong,” “Tan xiang,” and “Xiang zhong”. 2,2-diphenyl-1-picrylhydrazyl (DPPH); 2,2′-azinobis (3-ethylbenzothiazoline 6-sulfonate) (ABTS); and oxygen radical absorption capacity (ORAC) assays were used to assess the antioxidant activities of the PCs. Furthermore, we analyzed the anti-inflammatory action of these PCs using lipopolysaccharide (LPS)-induced RAW264.7 cells.

**Results:**

A total of 44 PCs were identified in the three cultivars. Of these, 17 PCs were previously unidentified in Chinese olive. Among the cultivars, the free phenolics (FPs) of “Tan xiang” showed the strongest antioxidant activity. All cultivars have shown significant inhibition of TNF-α and IL-6 production. Clustering correlation analysis showed galloyl-bis-HHDP-glucose and paeonol have significant anti-inflammatory ability in FPs. Quininic, galloylquinic acid, 4-hydroxycinnamic acid and gallic acid hexoside have shown significant inhibition of IL-6 production in BPs. Furthermore, gallic acid, catechin, syringic acid, and nobiletin exhibit negative correlation in FPs and positive correlation in BPs of cytokine production, while corilagin and methyl ellagic acid pentoside exhibited opposite correlation.

**Conclusion:**

In summary, this study contributed to the literature on PCs in Chinese olives and the potential health benefits of FPs and BPs.

## 1 Introduction

Polyphenols are ubiquitous secondary metabolites found in plants and exert potent beneficial effects on human health (1). There are more than 8,000 known phenolic compounds (PCs) existing in the form of free phenolics (FPs) and bound phenolics (BPs). FPs are present in free forms within the plant cell vacuole, while BPs are linked to the insoluble macromolecules in the cell wall via covalent bonds, hydrogen bonds, and hydrophobic interactions (2). The composition and content of FPs and BPs vary across plant species, resulting in varying pharmacological effects on the host plants. This divergence underscores the importance of investigating the function and distribution of PCs in different plant species (3).

Chinese olive (*Canarium album* L.) is a fusiform drupe fruit belonging to the *Burseraceae* family, originating in southeastern China. In China, the Chinese olive has a long history of edible and medicinal use, setting it apart from the European olive (*Olea europaea* L.), which is primarily used for olive oil extraction (4). In traditional Chinese medicine, Chinese olive is known as “Qing guo” and is believed to treat pharyngitis, faucitis, stomatitis, hepatitis, and toxicosis (5). Interestingly, in the Guangdong province, it is added to soup as a remedy for pharyngitis.

Chinese olive is a rich source of phytonutrients, particularly PCs. As one of the 110 medicinal foods in China, Chinese olive contains higher free phenolic levels (300 mg/100 g fresh weight) than grapes, apples, pears, and other fruits and also ranks first among 68 Chinese medicinal materials in terms of phenolic content (6). Despite being rich in FPs (7, 8), there is a paucity of studies comparing the PCs profiles of different Chinese olive cultivars, especially the bound phenolic fraction.

Phenolic compounds can modulate the expression of several pro-inflammatory genes (such as those encoding lipoxygenase, nitric oxide synthases, and cyclooxygenase), thereby attenuating the inflammatory signaling pathways (9). Additionally, they exhibit antioxidant effects by suppressing reactive oxygen species (ROS)-mediated inflammation (10). Notably, the anti-inflammatory activities of PCs can vary depending on their composition, even when the overall content remains constant (11). However, the factors responsible for the varying activities of PCs and the bioactivities of different PCs in different forms have not yet been well explored. This gap in knowledge hinders the optimal selection of functional cultivars to develop desired products.

This study aimed to identify and quantify the FPs and BPs present in three commercially important Chinese olive cultivars. The antioxidant activity was measured by 2,2-diphenyl-1-picrylhydrazyl (DPPH), 2,2′-azinobis (3-ethylbenzothiazoline 6-sulfonate) (ABTS) and oxygen radical absorption capacity (ORAC) methods. The anti-inflammatory activities of the PCs were assessed in lipopolysaccharide (LPS)-induced RAW264.7 cells. This study supplements the phenolic compound database of Chinese olives. In addition, our results might help enhance the functionality, nutritional value, and health benefits of foods by adding the characterized PCs as antioxidants and anti-inflammatory additives.

## 2 Materials and methods

### 2.1 Chemical reagents

The standard PC reference materials (Quinic acid, Gallic acid, Vanillic acid, Syringic acid, Protocatechuic acid, 4-Hydroxybenzoic acid, Chlorogenic acid, Esculetin, Caffeic acid, 4-Hydroxycinnamic acid, Ferulic acid, Isoferulic acid, Coumaric acid, Genistin, Genistein, Hyperoside, Astragalin, Isoquercitrin, Quercetin, Kaempferol, (-)-Gallocatechin, Procyanidin B1, Catechin, Procyanidin B2, Epicatechin, Corilagin, Paeonol, Ellagic acid, Syringaldehyde, Rutin) were purchased from Yuanye Scientific Co. (Shanghai, China). Analytical-grade chemicals were purchased from Kermel Scientific Co. (Tianjin, China). 2,2-diphenyl-1-picrylhydrazyl (DPPH); 2,2′-azinobis-(3-ethylbenzothiazoline-6-sulfonic acid) (ABTS); 2,2′-azobis (2-amidinopropane) dihydrochloride (AAPH); and 5,7,8-tetramethylchroman-2-carboxylic acid (Trolox) were purchased from Sigma–Aldrich (Shanghai, China). Macrophage RAW264.7 cells were purchased from Jinan University (Guangzhou, China). Dulbecco's Modified Eagle Medium (DMEM) was obtained from Procell Life Science & Technology Co., Ltd. (Wuhan, China). Penicillin-streptomycin, fetal bovine serum (FBS), Cell Counting Kit-8 (CCK-8), and nitric oxide (NO) kit were purchased from New Cell & Molecular Biotech (Suzhou, China), ExCell Bio (Suzhou, China), Yeasen Biotechnology Co., Ltd. (Shanghai, China); and Beyotime Biotechnology (Shanghai, China); respectively. Mouse TNF-α and IL-6 ELISA kits were acquired from NeoBioscience Technology (Shenzhen, China).

### 2.2 Sample preparation and extraction of free and bound phenolics

Mature fruits were obtained from the Chaoshan region of the Guangdong province, China. The collected fruits belonged to three cultivars: “Na zhong” (moisture content: 30.07%), “Tan xiang” (moisture content: 14.59%), and “Xiang zhong” (moisture content: 13.94%). All fruits were washed, cored, sliced, and stored at −80°C for 12 h. The frozen slices were dried using a vacuum lyophilizer (Christ, Osterode am Harz, Germany), powdered, and kept at −20°C before subsequent analyses.

The PCs were extracted using the method introduced by Li et al. (12).

#### 2.2.1 FPs extraction

Three grams of each powdered sample was treated thrice with 30 mL hexane to remove lipids. Then, the sample was extracted with 90 mL of 80% methanol (1% HCl) and ultrasonicated for 30 min. The solution was then centrifuged at 8,000 rpm for 15 min at 4°C, and the supernatant was collected (ultrasonic extraction was repeated twice).

#### 2.2.2 BPs extraction

The residues obtained after the centrifugation step during FP extraction were treated with 90 mL of 3 M NaOH (10 mM Ethylenediaminetetraacetic acid, 1% L-Ascorbic acid) for 4 h under continuous oscillation. Subsequently, 6 M HCl was added to the mixtures to adjust the pH to 2.0. Each solution was centrifuged at 8,000 rpm for 15 min at 4°C, and the supernatant was collected. Each supernatant was extracted with 90 mL of 3 M HCl for 1 h at 85°C. Then, 6 M NaOH was added to the extracted residue to adjust the pH to 2.0. The solution was then centrifuged at 8,000 rpm for 15 min at 4°C, and the supernatant was collected. Subsequently, the two parts of extracts obtained by acid and alkaline hydrolysis were combined.

The FPs- and BPs-containing supernatants were combined with equal parts of ethyl acetate to extracted PCs (extraction thrice), evaporated using a rotary vacuum evaporator (BUCHI, China), and resolubilized with methanol. Those were freeze-dried for further analyses, and called them FPs and BPs, separately.

### 2.3 Analyses of total phenolic content and total flavonoid content

Sample extracts of FPs and BPs were dissolved by methanol for analyses. TPC was measured using the method introduced by You et al. (13). Briefly, 20 μL of sample solution was mixed with 100 μL of 0.2 N Folin–Ciocalteu reagent in 96-well plates and incubated for 10 min. Then, 100 μL of 10% Na_2_CO_3_ was added to each well, and the solutions were incubated for 30 min at room temperature. Finally, the absorbance of each solution was measured at 765 nm using a microplate reader (Molecular Devices, United States). The TPC was expressed as milligrams of gallic acid equivalents per gram of dried weight of Chinese olive (mg GAE/g DW; ABS concentration curve: *y* = 0.0058x + 0.0164 *R*^2^ = 0.9991).

TFC was measured using the method introduced by Yang et al. (14). Briefly, Sample extracts were dissolved by methanol, 25 μL of sample solution was mixed with 110 μL of 0.66 M NaNO_2_ in 96-well plates and incubated for 5 min. Then, 10 μL of 0.75 M AlCl_3_ was added to each well, and the plates were allowed to stand for 6 min. Then, 100 μL of 0.5 M NaOH was added to each well. Finally, the absorbance of the solutions was measured at 510 nm using the microplate reader. The TFC was expressed as milligrams of rutin equivalents per gram of dried weight of Chinese olive (mg RE/g DW; ABS concentration curve: *y* = 0.0005x – 0.0037 *R*^2^ = 0.9997).

### 2.4 Ultra-high performance liquid chromatography coupled with hybrid quadrupole-orbitrap/mass spectrometry (UPLC-Q-Exactive Orbitrap/MS) analysis

Sample extracts of FPs and BPs were dissolved by methanol for detection. The PCs were quantified using the UltiMate 3000 liquid chromatographic system (Thermo Fisher Scientific, China) with an ACQUITY UPLC BEH C18 column (2.1 mm × 100 mm × 1.7 μm; Waters, USA). The elution was done using acetonitrile as solvent A and water with 0.1% formic acid as solvent B. The PCs were identified and quantified using Q-Exactive Orbitrap/MS (Thermo Fisher Scientific, China) equipped with an electrospray ionization (ESI) source. The gradient was formed as follows: 0–3 min 95–85% B, 3–11 min 85–70% B, 11–15 min 70–50% B, 15–21 min 50–10% B, 21–22 min 10–95% B. The flow rate was 150 μL/min with a sample injection volume of 2 μL, and the column temperature was equilibrated to 20°C (14). MS experiments were conducted in positive and negative scan ionization mode interfaces. The protocol was set as follows: Auxiliary gas (N_2_), 10 arb; sheath gas (N_2_), 35 arb; capillary voltage, 3,200 V; capillary temperature, 320°C; scan range, m/z 70–1,500. Before the analysis, an external calibration was performed to determine the accuracy of the masses measured. The Trace Finder software (Thermo Fisher Scientific, version 4.0) was used to analyze the data. The compounds were identified by comparing with analytical standards or comparing them against the exact masses, MS/MS mass spectra, and molecular formulas obtained from the available literature. When the standard was unavailable, the External calibration curve corresponding to a structure-related PC was used to quantify the compound tentatively. Supplementary material II summarize the parameters of the method used for each standard: calibration curve (*y* = *a* + *bx*), *R*^2^ and linear range.

### 2.5 Analyses of antioxidant capacity assay

Antioxidant activities of PCs were assessed based on their DPPH and ABTS scavenging activities and oxygen radical absorbance capacity (ORAC). The sample extracts of FPs and BPs were dissolved by methanol for detection. The DPPH scavenging activities of the PCs were assessed using the method introduced by Cheng et al. (15). Briefly, 100 μL of sample solution was mixed with 100 μL of 0.5 mM DPPH and incubated for 30 min in the dark. The microplate reader (Molecular Devices, United States) was used for multimode spectrophotometric detection of the solutions at 517 nm.

For the ABTS assay, we first prepared the ABTS solution (16). Briefly, 7 mM ABTS was mixed with 2.45 mM potassium persulfate at room temperature (23°C) in the dark for 16 h. The prepared solution was diluted with 80% ethanol to an absorbance of 0.700 ± 0.050 at 734 nm to obtain the ABTS working solution. Then, 200 μL of the ABTS working solution was mixed with 20 μL of sample solution, and the solution was incubated for 10 min. The absorbance of the solution was measured at 734 nm using the microplate reader.

For ORAC assay (17), a total of 100 μL of diluted fluorescein sodium (95.6 nM) solution was mixed with 25 μL of each sample in a black 96-well plate and incubated at 37°C for 15 min. Subsequently, 75 μL of 150 mM AAPH solution was added to each well, and the fluorescence intensity was measured every 2 min for 2 h via the scheduled recording function of a fluorometer with excitation at 485 nm and emission at 530 nm.

All results of antioxidant assay were expressed as mg Trolox equivalents per gram of dry weight (mg TE/g DW) [ABS concentration curves for (1) DPPH: *y* = 0.6367x + 1.1433 *R*^2^ = 0.9994; (2) ABTS: *y* = 0.2068x – 0.384 *R*^2^ = 0.9991; and (3) ORAC: *y* = 285588x + 25893 *R*^2^ = 0.9997].

### 2.6 Anti-inflammatory activity assay

#### 2.6.1 Cell culture

RAW264.7 cells were cultured in DMEM supplemented with 10% (v/v) FBS and 1% (v/v) penicillin-streptomycin. The culture was incubated in a humidified atmosphere (37°C and 5% CO_2_) until the cells reached 80% confluency.

#### 2.6.2 Cell viability analysis

The cells were sub-cultured to a concentration of 3.0 × 10^4^ cells/mL (100 μL culture in each well of a 96-well plate) and incubated for 24 h. Then, we measured cell viability using the CCK-8 assay. Briefly, sample extracts of FPs and BPs were dissolved in not exceeding 0.1% DMSO and diluted immediately before cell treatments. Varying concentrations of culture medium containing sample solution (0, 25, 50, 100, and 200 μg TPC/mL) were added to different wells, and the cultures were incubated for 24 h. Culture media were replaced with 100 μL of fresh DMEM and 10 μL of CCK-8 solution. The plate was then incubated for 2 h. Finally, the absorbance of the culture in each well was measured at 450 nm using the microplate reader.

#### 2.6.3 Detection of inflammatory mediators

RAW264.7 cells were stimulated with LPS to induce inflammation. The cells were then cultured in 24-well plates at 37°C (concentration per well: 1.0 × 10^5^ cells/mL, total volume/well: 500 μL) for 24 h. Then, the culture medium was discarded, phenolic extraction (suitable concentrations were found from CCK-8 assay) and LPS (1 μg/mL) (three biological replicates per concentration). After incubation for 24 h, the supernatant after centrifugation was collected and subjected to NO, TNF-α, and IL-6 assays according to the manufacturers' protocols.

### 2.7 Statistical analysis

Partial least squares discriminant analysis (PLS-DA) was applied to establish the classification model among the three cultivars. The relationships in terms of bioactivities (antioxidant and anti-inflammation activity) and PCs were visualized using a Clustering correlation heatmap with signs. PLS-DA and Clustering correlation heatmap were carried out using the OmicStudio tools at https://www.omicstudio.cn/tool. Data were presented as the means ± standard deviation for each experimental group. GraphPad Prism 8.0 software was used to analyze the statistical significance based on the One-way Analysis of Variance (ANOVA) followed by Tukey's *post-hoc* test. Differences with *p* < 0.05 were considered statistically significant.

## 3 Results and discussion

### 3.1 TPC and TFC in Chinese olive cultivars

**Figure 1:**
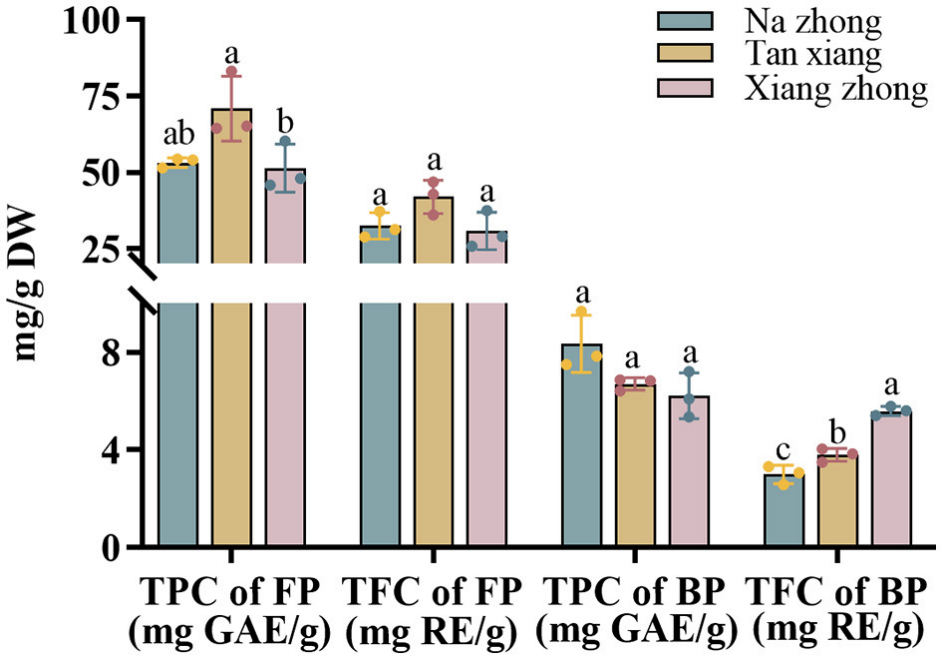
Total phenolic contents and total flavonoid contents of free and bound phenolic fractions extracted from three cultivars of dry Chinese olive. TPC, total phenolic content; TFC, total flavonoid content; FP, free phenolic; BP, bound phenolic. Values represent mean ± standard deviation (*n* = 3). Different lowercase letters in the same column indicate significant differences (*p* < 0.05) between Chinese olive cultivars.

The TPC and TFC of the extracts from the three olive cultivars are shown in Figure 1. While measuring TPC, we observed that the “Tan xiang” cultivar exhibited the highest free phenolic levels (70.94 ± 10.59 mg GAE/g DW), followed by “Na zhong” and “Xiang zhong”. We observed significant differences in the free phenolic levels of “Tan xiang” and “Xiang zhong”. On the other hand, the “Na zhong” cultivar exhibited the highest bound phenolic levels (8.34 ± 1.17 mg GAE/g DW), followed by “Tan xiang” and “Xiang zhong”. Bound phenolic levels accounted for 9% to 16% of the TPC. The three cultivars did not differ significantly in terms of the proportion of BPs in the TPC.

For all three cultivars, FPs accounted for 59–61% of TFC, with no significant differences among the cultivars in terms of free phenolic proportions (Figure 1). Furthermore, while measuring TFC, the “Tan xiang” cultivar harbored the highest of TFC level (32.50 ± 4.33 mg RE/g DW), followed by “Na zhong” and “Xiang zhong”. The three cultivars differed significantly in terms of the proportion of BPs in the TFC fraction (*p* < 0.05).

For the three cultivars, the proportions of FPs measured in the TPC and TFC fraction in the present study were significantly lower than those reported by Guo et al. (18) and Liu et al. (6) but similar to reported by Chang et al. (19). In contrast, the content of free phenolic can be influenced by factors such as temperature, mass-to-solvent ratio, and pre-treatment (particle size and antioxidant) (20, 21). Traditional extraction solvents include methanol, ethanol, acetone and water, but their extraction yields may not be useful for different fruits. Belwal et al. (22) found that higher temperatures and higher concentrations of methanol were better than other solvents in the extraction of *Berberis asiatica* fruit, whereas the best extraction conditions to peach were 60% acetone from Mokrani and Madani (23). Additionally, the cultivar, origin, and other factors related to fruit can also affect the content (24). To the best of our knowledge, this study was the first to explore the bound phenolic content in *C. album* L., showing the insoluble bound forms of PCs as a significant part of the TPC in vegetables and fruits (25). In comparison to the bound phenolic ratio TFC of *Olea europaea* L. (26) ranging from 1 to 21% for different extractants, Farag RS (27) reported 18% for two cultivars and Alu'datt MH (28) reported 33%. BPs are possible health-benefitting agents because they can act as carriers of phenolics, reaching the colon via dietary fiber and potentially exerting antioxidant, antimicrobial, and anti-inflammatory effects (29).

### 3.2 Identification and quantification of individual extractable phenolic compounds in Chinese olive cultivars

Forty-four PCs were detected in the three cultivars using the UPLC-Q-Exactive Orbitrap/MS analysis (Supplementary Table 1; Supplementary Figure 1). Of these, 29 were identified based on their standards (Supplementary Table 2), and the other peaks were tentatively identified based on the exact masses, MS/MS spectra, and molecular formulas extracted from previous literature. All the detected PCs were categorized into seven classes: hydroxybenzoic acids, hydroxycinnamic acids, isoflavones, flavones, flavonols, flavanols, and other polyphenols and derivatives.

In fruits and vegetables, phenolic acids usually exist in the free form, distinguishable as hydroxybenzoic acids and hydroxycinnamic acid (30). Table 1 lists the 13 phenolic acids, 14 flavonoids, and 11 other polyphenols identified in the free phenolic fraction of *C. album* L. The free phenolic fraction was found to be notably rich in phenolic acids, accounting for 65–79% of the TPC. Quinic acid, gallic acid, syringic acid, protocatechuic acid, 4-hydroxybenzoic acid, chlorogenic acid, 4-hydroxycinnamic acid, isoferulic acid, and cinnamic acid were detected in the free phenolic fraction of all three Chinese olive cultivars. Quinic acid, with levels ranging from 1229.69–1381.07 mg/100 g DW, emerged as the most dominant phenolic acid in Chinese olive, followed by galloylquinic acid and brevifolincarboxylic acid. The MS/MS fragmentation ions at m/z 191.05504 and 169.01312 of peak ID 2 (Table 1) indicate the presence of galloylquinic acid. The loss of 162 mass units for peak IDs 3 and 5 (331.06583–169.01259 and 329.08701–167.03391, respectively) suggested the existence of a hexoside, leading to the tentative identification of gallic acid hexoside and vanillic acid hexoside, respectively. Notably, vanillic acid hexoside was exclusively found in the free phenolic fraction; however, vanillic acid was not detected in Chinese olives. Furthermore, chlorogenic acid was also exclusively present in the free phenolic fraction, with significantly varying quantities across the three cultivars.

**Table 1:** Quantification of free phenolic (FPs) in *Canarium album* L.

**ID**	**Tentative compound**	**RT (min)**	**Molecular formula**	***Canarium album*** **L. (mg/100 g DW)**		
				**“Na zhong”**	**“Tan xiang”**	**“Xiang zhong”**
**Hydroxybenzoic acids and derivatives**						
1	Quinic acid	1.67	C7H12O6	1229.69 ± 97.92^a^	1278.65 ± 133.28^a^	1381.07 ± 211.15^a^
2	Galloylquinic acid^*^	2.66	C14H16O10	296.50 ± 62.78^a^	198.06 ± 19.56^a^	283.51 ± 72.44^a^
3	Gallic acid hexoside^*^	2.70	C13H16O10	15.04 ± 1.47^b^	29.14 ± 6.01^a^	22.67 ± 2.71^ab^
4	Gallic acid	3.91	C7H6O5	9.62 ± 1.56^a^	13.13 ± 3.97^a^	13.41 ± 2.52^a^
5	Vanillic acid hexoside^*^	4.01	C14H18O9	8.18 ± 1.20^b^	13.24 ± 0.82^a^	10.70 ± 2.83^ab^
6	Protocatechuic acid	5.76	C7H6O4	0.29 ± 0.12^b^	0.58 ± 0.08^a^	0.41 ± 0.11^ab^
7	Brevifolincarboxylic acid^*^	6.31	C13H8O8	44.91 ± 12.93^b^	82.21 ± 8.01^a^	38.31 ± 3.70^b^
8	4-hydroxybenzoic acid	6.41	C7H6O3	0.51 ± 0.04^b^	1.27 ± 0.17^a^	0.65 ± 0.09^b^
9	Syringic acid	7.21	C9H10O5	0.23 ± 0.08^a^	0.33 ± 0.04^a^	0.27 ± 0.05^a^
Total (mg/100 g DW)				1604.98 ± 134.09^a^ (77.11%)	1616.62 ± 170.19^a^ (64.76%)	1750.99 ± 229.33^a^ (78.59%)
**Hydroxycinnamic acids and derivatives**						
10	Chlorogenic acid	6.06	C16H18O9	0.23 ± 0.01^c^	0.92 ± 0.07^a^	0.64 ± 0.09^b^
11	4-hydroxycinnamic acid	8.78	C9H8O3	0.01 ± 0.01^b^	0.05 ± 0.02^a^	ND
12	Isoferulic acid	10.21	C10H10O4	0.23 ± 0.06^b^	0.58 ± 0.15^a^	0.24 ± 0.05^b^
13	Cinnamic acid	15.60	C9H8O2	0.81 ± 0.27^b^	2.85 ± 0.68^a^	1.36 ± 0.21^b^
Total (mg/100 g DW)				1.28 ± 0.34^b^ (0.06%)	4.40 ± 0.85^a^ (0.18%)	2.25 ± 0.25^b^ (0.10%)
**Isoflavones and derivatives**						
14	Genistin	10.16	C21H20O10	0.03 ± 0.01^a^	0.04 ± 0.01^a^	ND
Total (mg/100 g DW)				0.03 ± 0.01^a^ (0%)	0.04 ± 0.01^a^ (0%)	0%
**Flavones and derivatives**						
15	Hyperoside	9.12	C21H20O12	45.52 ± 5.29^b^	102.59 ± 16.91^a^	47.00 ± 4.29^b^
16	Astragalin	10.12	C21H20O11	8.72 ± 3.22^b^	27.67 ± 2.67^a^	7.96 ± 1.72^b^
17	Nobiletin^*^	16.85	C21H22O8	0.05 ± 0.02^b^	0.40 ± 0.06^a^	0.23 ± 0.17^ab^
Total (mg/100 g DW)				54.30 ± 8.18^b^ (2.61%)	130.66 ± 18.73^a^ (5.23%)	55.19 ± 5.96^b^ (2.48%)
**Flavonols and derivatives**						
18	Isoquercitrin	9.27	C21H20O12	35.57 ± 4.35^b^	82.52 ± 13.92^a^	36.70 ± 3.54^b^
19	Kaempferol 3-O-glucoside^*^	10.00	C21H20O11	14.26 ± 5.20^b^	44.80 ± 4.35^a^	13.02 ± 2.75^b^
20	Quercetin	14.56	C15H10O7	ND	2.28 ± 0.67^a^	ND
21	Kaempferol	16.32	C15H10O6	ND	0.39 ± 0.15^a^	ND
Total (mg/100 g DW)				49.83 ± 9.13^b^ (2.39%)	129.99 ± 17.81^a^ (5.21%)	49.72 ± 6.01^b^ (2.23%)
**Flavanols and derivatives**						
22	Gallocatechin	4.30	C15H14O7	0.53 ± 0.13^b^	1.00 ± 0.02 ^a^	0.54 ± 0.09 ^b^
23	Procyanidin B1	5.44	C30H26O12	0.82 ± 0.05 ^b^	1.54 ± 0.40^a^	1.03 ± 0.23^ab^
24	Epigallocatechin^*^	5.55	C15H14O7	0.13 ± 0.01^a^	0.12 ± 0.01^a^	0.03 ± 0.01^b^
25	Catechin	5.88	C15H14O6	2.45 ± 0.98^a^	3.90 ± 0.19^a^	3.41 ± 1.04^a^
26	Procyanidin B2	6.57	C30H26O12	0.78 ± 0.05^b^	1.40 ± 0.14^a^	0.22 ± 0.02^c^
27	Epicatechin	6.95	C15H14O6	0.85 ± 0.20^b^	1.49 ± 0.16^a^	0.17 ± 0.07^c^
Total (mg/100 g DW)				5.61 ± 1.44^b^ (0.27%)	9.44 ± 0.79^a^ (0.38%)	5.43 ± 1.41^b^ (0.24%)
**Other polyphenols**						
28	Geraniin isomers^*^	6.71	C41H28O27	30.76 ± 1.40^b^	106.45 ± 6.11^a^	39.80 ± 4.96^b^
29	Corilagin (1-O-galloyl-3,6-(R)-HHDP-β-d-glucose)	6.79	C9H10O4	203.00 ± 6.14^b^	271.02 ± 45.81^a^	179.86 ± 4.70^b^
30	Galloyl-bis-HHDP-glucose^*^	6.86	C41H28O26	3.85 ± 0.36^b^	3.64 ± 0.19^b^	8.08 ± 1.02^a^
31	Geraniin^*^	7.78	C41H28O27	23.69 ± 0.99^b^	33.86 ± 2.20^a^	25.47 ± 9.11^ab^
32	Ellagic acid pentoside^*^	7.85	C19H14O12	47.09 ± 3.61^b^	114.63 ± 10.47^a^	48.26 ± 3.85^b^
33	Ellagic acid glucuronoside^*^	8.37	C20H14O14	15.74 ± 0.30^a^	ND	15.41 ± 0.06^a^
34	Methyl-ellagic acid glucuronoside^*^	8.67	C21H16O14	5.37 ± 3.01^a^	6.32 ± 0.87^a^	4.65 ± 1.21^a^
35	Ellagic acid	8.84	C14H6O8	27.30 ± 2.23^b^	66.12 ± 15.05^a^	40.30 ± 2.90^b^
36	Methyl ellagic acid pentoside^*^	9.83	C20H16O12	8.11 ± 3.50^a^	2.52 ± 0.38^b^	1.71 ± 0.10^b^
37	Syringaldehyde	10.75	C9H10O4	0.38 ± 0.04^a^	0.36 ± 0.02^a^	0.41 ± 0.05^a^
38	Paeonol	17.28	C9H10O3	0.05 ± 0.04^a^	0.41 ± 0.20^a^	0.36 ± 0.15^a^
Total (mg/100 g DW)				365.33 ± 18.99^b^ (17.55%)	605.33 ± 80.58^a^ (24.25%)	364.31 ± 8.44^b^ (16.35%)
Total PC (mg/100 g DW)				2,081.36 ± 131.54^a^	2,496.48 ± 285.13^a^	2,227.89 ± 226.04^a^

Values represent the mean ± standard error (n = 3). The different lowercase letters between each row indicate significant differences (p < 0.05) between the Chinese olive cultivars. Quantification was reported in mg/100 g DW.

RT, retention time; ND, not detected.

^*^Galloylquinic acid, gallic acid hexoside and brevifolincarboxylic acid were quantified in gallic acid equivalents. Vanillic acid hexoside was quantified in vanillic acid equivalents. Nobiletin was quantified in hyperoside equivalents. Kaempferol 3-O-glucoside was quantified in kaempferol equivalents. Epigallocatechin was quantified in gallocatechin equivalents. Geraniin isomers, galloyl-bis-HHDP-glucose, geraniin, ellagic acid pentoside, ellagic acid glucuronoside, methyl-ellagic acid glucuronoside, and methyl ellagic acid pentoside were quantified in ellagic acid equivalents.

The “Tan xiang” cultivar exhibited significantly higher levels of flavones, flavanols, and their derivatives than the other two cultivars (*p* < 0.05). Although genistin was detected (8), its limited quantity precluded quantification.

Apart from phenolic acids and flavonoids, we identified 11 non-flavonoid polyphenols in Table 1. Peak ID 29 was identified as corilagin (galloyl-HHDP-glucose) with a deprotonated ion at m/z 633.07343 [M-H]^−^ and exhibited MS/MS fragments at m/z 300.99860 following the loss of an HHDP-group. Similarly, peak ID 30 displayed a molecular ion peak at m/z 935.07928 [M-H]^−^. Moreover, the losses of 15 mass units (315.01434–300.99850), 176 mass units (491.04654–315.01434), and 132 mass units (447.05658–315.01450) were indicative of methyl, glucuronoside, and pentoside groups, leading to the identification of peak IDs 32, 34, and 36 as ellagic acid pentoside, methyl-ellagic acid glucuronoside, and methyl ellagic acid pentoside, respectively (31, 32). Based on distinct retention times, we identified a geraniin isomer and geraniin at peak IDs 28 (m/z 951.07397) and 31 (m/z 951.07397), respectively.

Table 2 illustrates the 14 phenolic acids, 8 flavonoids, and 8 other polyphenols identified in the bound phenolic fraction. The bound phenolic fraction of the “Tan xiang” cultivar contained significantly higher levels of hydroxybenzoic acids and their derivatives than the bound phenolic fractions of other cultivars. The bound phenolic fraction of “Na zhong” cultivar harbored higher levels of hydroxycinnamic, isoflavones, and other polyphenols and their derivatives than the bound phenolic fractions of the other two cultivars. Notably, caffeic acid, esculetin, ferulic acid, genistein, amentoflavone, and dihydrokaempferol were exclusively found in the bound phenolic fraction of the cultivars.

**Table 2:** Quantification of bound phenolic (BPs) in *Canarium album* L.

**ID**	**Tentative compound**	**RT (min)**	**Molecular formula**	***Canarium album*** **L. (mg/100 g DW)**		
				**“Na zhong”**	**“Tan xiang”**	**“Xiang zhong”**
**Hydroxybenzoic acids and derivatives**						
1	Quinic acid	1.67	C7H12O6	3.67 ± 0.44^b^	23.49 ± 5.40^a^	1.44 ± 0.32^b^
2	Galloylquinic acid^*^	2.66	C14H16O10	0.79 ± 0.07^a^	0.92 ± 0.55^a^	0.15 ± 0.01^a^
3	Gallic acid hexoside^*^	2.70	C13H16O10	0.02^a^	0.03 ± 0.01^a^	0.01^a^
4	Gallic acid	3.91	C7H6O5	8.56 ± 1.04^a^	10.47 ± 0.87^a^	13.24 ± 4.12^a^
5	Protocatechuic acid	5.76	C7H6O4	0.24 ± 0.13^a^	0.17 ± 0.04^a^	0.20 ± 0.04^a^
6	Brevifolincarboxylic acid^*^	6.31	C13H8O8	0.36 ± 0.06^a^	0.68 ± 0.34^a^	0.29 ± 0.02^a^
7	4-hydroxybenzoic acid	6.41	C7H6O3	1.60 ± 0.43^a^	1.26 ± 0.17^a^	1.20 ± 0.21^a^
8	Syringic acid	7.21	C9H10O5	0.99 ± 0.02^a^	0.93 ± 0.06^a^	0.95 ± 0.03^a^
Total (mg/100 g DW)				16.23 ± 1.22^b^ (34.18%)	37.96 ± 4.71^a^ (67.49%)	17.48 ± 4.60^b^ (56.82%)
**Hydroxycinnamic acids and derivatives**						
9	Caffeic acid	6.81	C9H8O4	0.19 ± 0.09^a^	0.12 ± 0.02^a^	0.13 ± 0.04^a^
10	Esculetin	7.05	C9H6O4	0.02^a^	ND	ND
11	4-hydroxycinnamic acid	8.78	C9H8O3	0.14 ± 0.02^ab^	0.16 ± 0.01^a^	0.08 ± 0.04^b^
12	Ferulic acid	9.53	C10H10O4	0.39 ± 0.33^a^	0.16 ± 0.04^a^	0.11 ± 0.04^a^
13	Isoferulic acid	10.21	C10H10O4	5.03 ± 1.38^a^	0.87 ± 0.19^b^	0.75 ± 0.16^b^
14	Cinnamic acid	15.60	C9H8O2	1.42 ± 0.08^a^	0.60 ± 0.34^ab^	0.86 ± 0.04^b^
Total (mg/100 g DW)				7.14 ± 1.22^a^ (15.04%)	1.91 ± 0.55^b^ (3.40%)	1.94 ± 0.18^b^ (6.30%)
**Isoflavones and derivatives**						
15	Genistin	10.16	C21H20O10	0.38 ± 0.12^a^	0.17 ± 0.08^a^	0.19 ± 0.05^a^
16	Genistein	16.02	C15H10O5	0.85 ± 0.07^a^	0.31 ± 0.10^b^	0.44 ± 0.13^b^
Total (mg/100 g DW)				1.23 ± 0.12^a^ (2.59%)	0.48 ± 0.16^b^ (0.86%)	0.63 ± 0.17^b^ (2.04%)
**Flavones and derivatives**						
17	Hyperoside	9.19	C21H20O12	1.10 ± 1.44^a^	0.08 ± 0.06^a^	0.02 ± 0.01^a^
18	Nobiletin^*^	16.85	C21H22O8	0.72 ± 0.05^a^	0.30 ± 0.07^b^	0.30 ± 0.06^b^
19	Amentoflavone^*^	17.06	C30H18O10	0.61 ± 0.90^a^	1.30 ± 0.32^a^	1.14 ± 0.39^a^
Total (mg/100 g DW)				2.43 ± 1.17^a^ (5.12%)	1.68 ± 0.32^a^ (2.99%)	1.46 ± 0.45^a^ (4.74%)
**Flavonols and derivatives**						
20	Quercetin	14.56	C15H10O7	0.45 ± 0.18^b^	2.48 ± 0.34^a^	2.08 ± 0.79^a^
21	Dihydrokaempferol^*^	15.73	C15H12O6	0.03^a^	0.01^b^	ND
Total (mg/100 g DW)				0.48 ± 0.19^b^ (1.02%)	2.49 ± 0.34^a^ (4.43%)	2.08 ± 0.79^a^ (6.77%)
**Flavanols and derivatives**						
22	Catechin	5.88	C15H14O6	0.03 ± 0.01^b^	0.14 ± 0.01^b^	0.29 ± 0.09^a^
Total (mg/100 g DW)				0.03 ± 0.01^b^ (0.05%)	0.14 ± 0.01^b^ (0.24%)	0.29 ± 0.09^a^ (0.95%)
**Other polyphenols and derivatives**						
23	Geraniin isomers^*^	6.71	C41H28O27	0.53 ± 0.39^a^	0.24 ± 0.11^a^	0.15 ± 0.05^a^
24	Corilagin (1-O-galloyl-3,6-(R)-HHDP-β-d-glucose)	6.79	C9H10O4	0.80 ± 0.19^a^	0.74 ± 0.11^a^	0.63 ± 0.03^a^
25	Geraniin^*^	7.78	C41H28O27	0.16 ± 0.07^a^	0.12 ± 0.03^a^	0.10 ± 0.02^a^
26	Ellagic acid pentoside^*^	7.85	C19H14O12	1.04 ± 0.67^a^	0.69 ± 0.56^a^	0.37 ± 0.24^a^
27	Ellagic acid	8.84	C14H6O8	9.69 ± 1.06^a^	8.15 ± 3.84^a^	3.47 ± 0.19^b^
28	Methyl ellagic acid pentoside^*^	9.83	C20H16O12	0.47 ± 0.49^a^	0.09 ± 0.01^a^	0.08 ± 0.01^a^
29	Syringaldehyde	10.75	C9H10O4	6.78 ± 0.23^a^	1.25 ± 0.36^b^	1.50 ± 0.17^b^
30	Paeonol	17.28	C9H10O3	0.49 ± 0.21^a^	0.32 ± 0.15^a^	0.57 ± 0.25^a^
Total (mg/100 g DW)				19.94 ± 1.70^a^ (41.99%)	11.58 ± 4.82^b^ (20.59%)	6.88 ± 0.40^b^ (22.37%)
Total PC (mg/100 g DW)				47.49 ± 2.66^a^	56.25 ± 2.36^a^	30.77 ± 5.10^b^

Values represent the mean ± standard error (n = 3). The different lowercase letters between each row indicate significant differences (p < 0.05) between the Chinese olive cultivars. Quantification was reported in mg/100 g DW.

RT, retention time; ND, not detected.

^*^Galloylquinic acid, gallic acid hexoside and brevifolincarboxylic acid were quantified in gallic acid equivalents. Nobiletin and amentoflavone were quantified in hyperoside equivalents. Dihydrokaempferol was quantified in kaempferol equivalents. Geraniin isomers, geraniin, ellagic acid pentoside, and methyl ellagic acid pentoside were quantified in ellagic acid equivalents.

To the best of our knowledge, the presence of gallic acid hexoside, vanillic acid hexoside, syringic acid, caffeic acid, esculetin, 4-hydroxycinnamic acid, isoferulic acid, astragalin, nobiletin, gallocatechin, procyanidin B1, galloyl-bis-HHDP-glucose, paeonol, ellagic acid pentoside, methyl-ellagic acid glucuronoside, methyl ellagic acid pentoside, and syringaldehyde in Chinese olive was reported for the first time in the present study. In this study, 15 and 6 PCs were exclusively found in the free and bound phenolic fractions, respectively, suggesting the potentially different beneficial effects of these PCs (3).

### 3.3 Antioxidant capacity of Chinese olive cultivars

**Figure 2:**
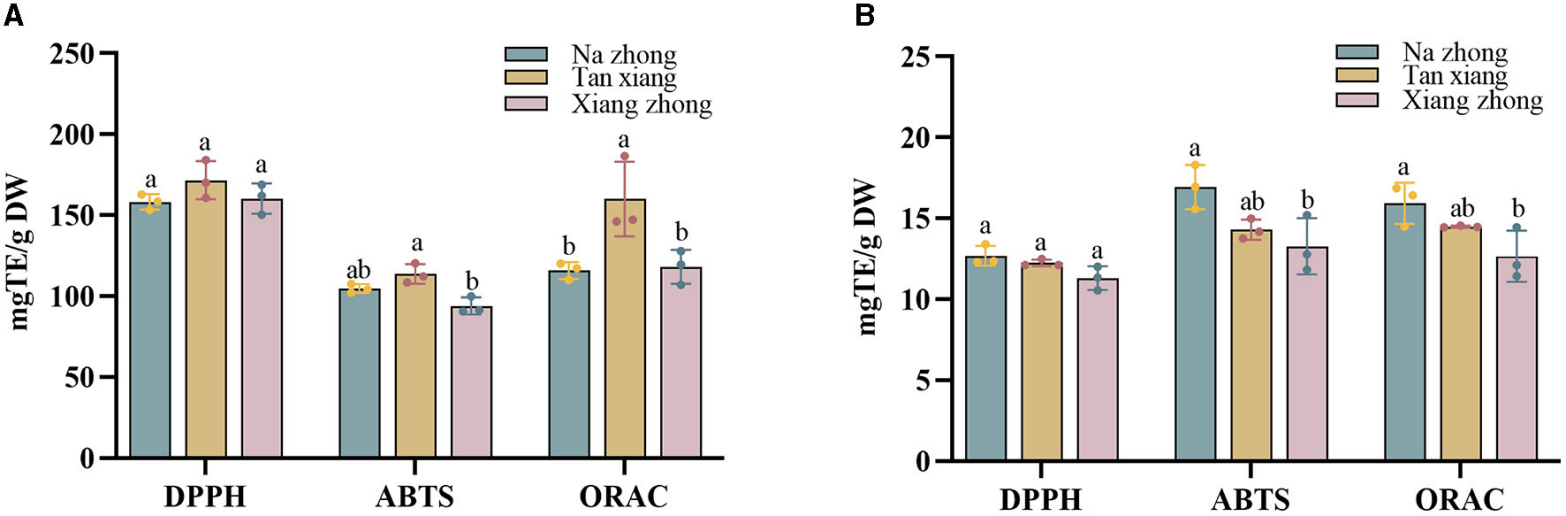
**(A)** The antioxidant capacity of free phenolics (FPs) in Chinese olive cultivars. **(B)** The antioxidant capacity of bound phenolics (BPs) in Chinese olive cultivars. Values represent mean ± standard deviation (*n* = 3). Different lowercase letters in the same column indicate significant differences (*p* < 0.05) between Chinese olive cultivars.

The antioxidant activities of the PCs present in Chinese olive cultivars were assessed using DPPH, ABTS, and ORAC assays (Figure 2).

The FPs present in the “Tan xiang” cultivar appeared slightly higher DPPH radical scavenging activity, followed by the FPs in “Xiang zhong” and “Na zhong” (158.20–171.62 mg TE/g DW). There was a trend toward in the BPs present, “Na zhong” appeared to have the highest DPPH radical scavenging activity, followed by the BPs in “Tan xiang” and “Xiang zhong” (11.30–12.69 mg TE/g DW). No significant differences were observed between the DPPH radical scavenging activities of FPs and BPs of any cultivar.

For the ABTS assay, the free phenolic fraction of the “Tan xiang” (104.66 ± 2.22 mg TE/g DW) cultivar exhibited higher ABTS scavenging activity, followed by “Na zhong” and “Xiang zhong,” (93.93–113.66 mg TE/g DW) with significant differences between “Xiang Zhong” and “Tan xiang”. The ORAC assay also showed that the free phenolic fraction of the “Tan xiang” cultivar significantly higher activity than the FPs of the other two cultivars. The BPs accounted for 11–14% of the ORAC value per gram of dry Chinese olive powder. The results of the ABTS and ORAC assays were consistent with those of the DPPH assay in terms of the antioxidant activity of BPs. Additionally, the bound phenolic fraction of “Na zhong” showed significantly higher ABTS scavenging activity and ORAC than the bound phenolic fraction of “Xiang zhong”.

These findings indicated that the “Tan xiang” cultivar exhibited the highest antioxidant activity among the three cultivars. Furthermore, compared to other Chinese medicinal herbs, the FPs in Chinese olive demonstrated a notably higher DPPH radical scavenging activity (16, 18). This discrepancy might be attributed to variations in experimental methods or procedures adopted across studies, though the overall antioxidant trends remained consistent. Similar findings have been reported in studies for other fruits, such as litchi (33) and blackberry (34). Moreover, the composition of the PCs in a food product crucially contributes to its antioxidant activity (35, 36).

### 3.4 Anti-inflammatory capacity of Chinese olive cultivars

**Figure 3:**
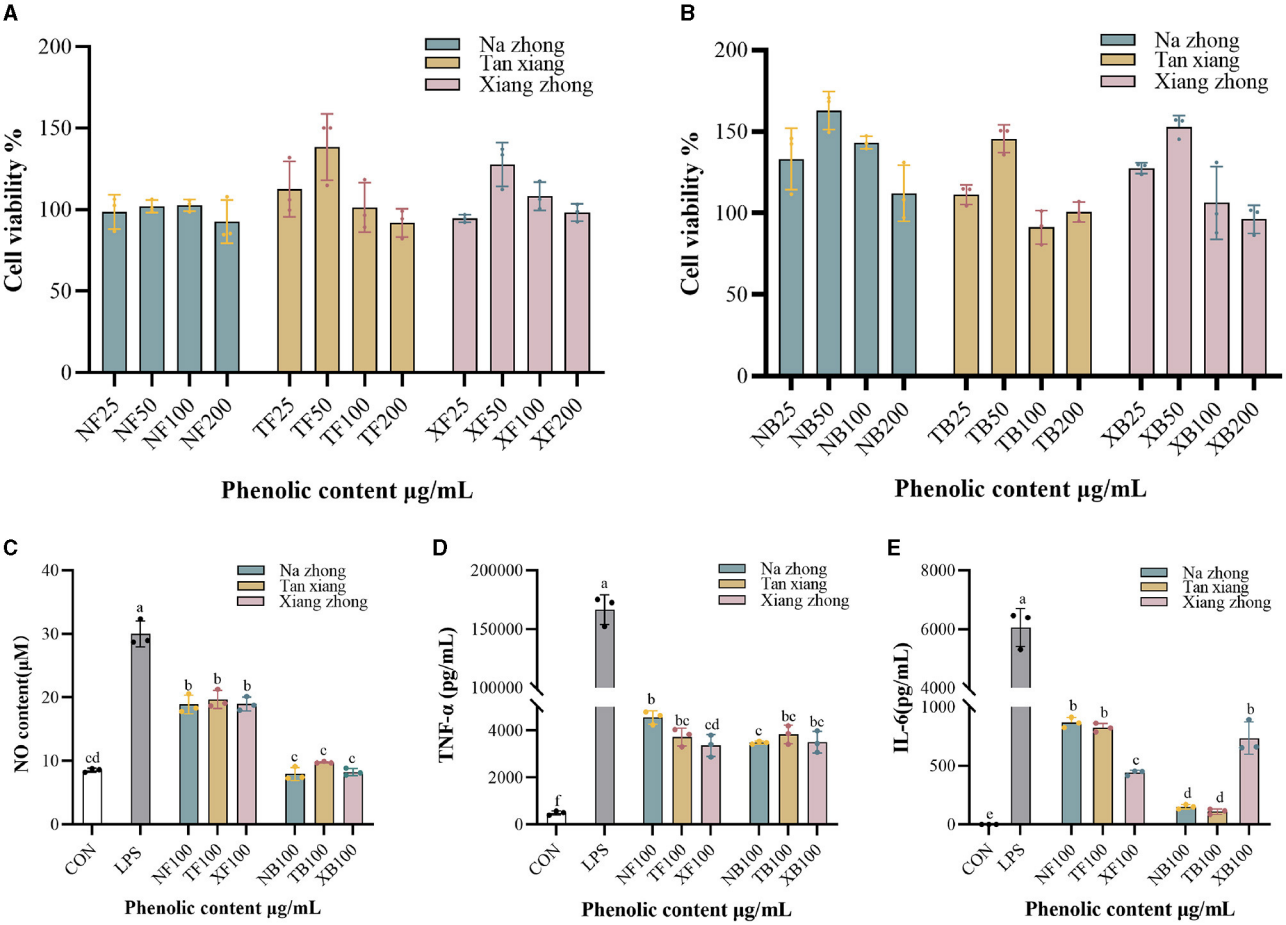
Anti-inflammatory effect of Chinese olive extracts on RAW264.7 cells. **(A)** Effects of free phenolics (FPs) on cell viability. **(B)** Effects of bound phenolics (BPs) on cell viability. **(C)** NO production. **(D)** TNF-α production. **(E)** IL-6 production. NF, free phenolics of “Na zhong”; TF, free phenolics of “Tan xiang”; XF, free phenolics of “Xiang Zhong”. NB, bound phenolics of “Na zhong”; TB, bound phenolics of “Tan xiang”; XB, bound phenolics of “Xiang Zhong”. Different lowercase letters in the same column indicate significant differences (*p* < 0.05) between Chinese olive cultivars.

We treated RAW264.7 cells with Chinese olive phenolic extracts to evaluate the cytotoxic effects and the anti-inflammatory activities of the extracts were shown in Figures 3A, B. No cytotoxicity was observed in cells treated with 25–200 μg/mL phenolic extracts. With increasing extract concentration, the cell viability first increased and then decreased, with maximum cell viability obtained after treatment with 50 μg/mL phenolic extract.

Recent studies have reported that Chinese olive fruit extracts possess anti-inflammatory properties (37). In the current study, we used NO, TNF-α, and IL-6 assays to explore the anti-inflammatory activity of the PCs in the three Chinese olive cultivars. As seen in Figures 3C–E, all intervention groups exhibited significantly low production of NO and cytokine secretion when compared to the LPS-stimulated control group (LPS group). It was found that the bound phenolic fraction of all cultivars exhibited no significant difference in NO production compared to the control group. Moreover, the TNF-α production of FPs was significantly decreased in “Xiang zhong” compared with “Na zhong”. However, no significant differences were observed in the BPs groups. Based on IL-6 assay (Figure 3E), the free phenolic fraction of “Xiang zhong” resulted in significantly lower levels levels of production than “Na zhong” and “Tan xiang”, whereas, this significance was reversed in the BPs groups. Interestingly, for the groups treated with “Na zhong” extracts, we observed a significantly higher suppression of cytokine production in the cells treated with BPs than the cells treated with FPs (*p* < 0.01). We also saw this suppression with the “Tan xiang” extracts in IL-6 production (*p* < 0.01).

Previous studies have indicated that the same PCs in different forms exhibit varying anti-inflammatory capacities (38). For instance, treatment with non-extractable phenolics led to a dose-dependent inhibition of LPS-induced NO production (39). Considering the potential limitations of anti-inflammatory exploration in cell models, it may be worthwhile to further explore and compare the activities of FPs and BPs in animal models.

### 3.5 Multivariate data analysis

**Figure 4:**
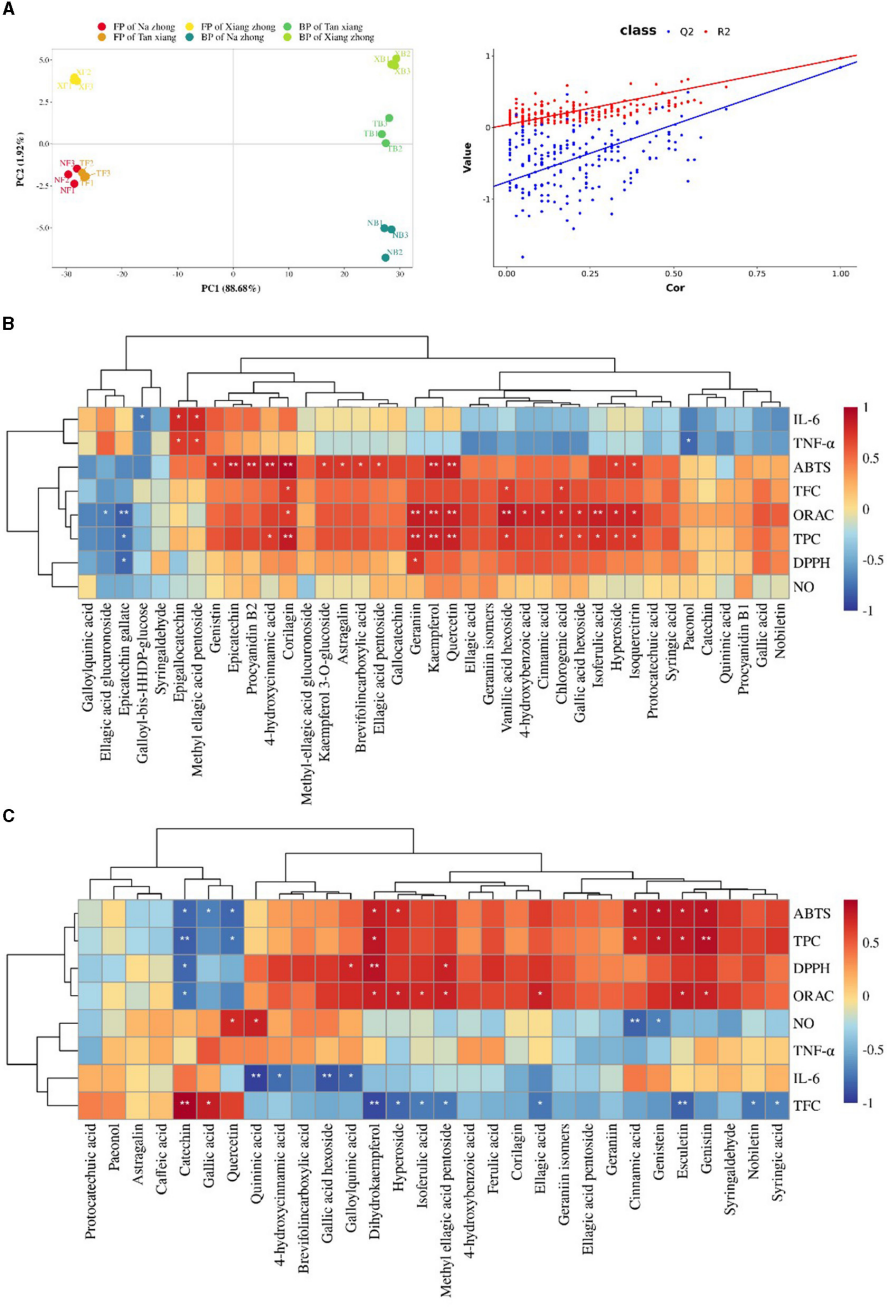
Multivariate analysis of the datasets of Chinese olive extracts. **(A)** The Partial least squares discriminant analysis (PLS-DA) score plot. NF, free phenolics of “Na zhong”; TF, free phenolics of “Tan xiang”; XF, free phenolics of “Xiang Zhong”. NB, bound phenolics of “Na zhong”; TB, bound phenolics of “Tan xiang”; XB, bound phenolics of “Xiang Zhong”. **(B)** Clustering correlation heatmap with signs in free phenolics. **(C)** Clustering correlation heatmap with signs in bound phenolics. Darker red and darker blue represent higher levels of positive and negative correlations, respectively. Significant correlations are marked with asterisks: *P < 0.05, **P < 0.01.

The (dis)similarities among the different cultivars were effectively visualized using the PLS-DA score plot and the clustering heatmap. The PLS-DA score plot shown in Figure 4A depicts the grouping of free and bound phenolic extracts based on the cultivars, with the most influential variables contributing to 90.60% of the total variance. The *R*^2^ (0.966) and Q2 (0.841) values demonstrated the model's stability and predictive power. In this study, a screening criterion of variable importance in projection (VIP) > 1 was used. It revealed several potentially important compounds, including isoquercitrin, kaempferol 3-O-glucoside, vanillic acid hexoside, methyl-ellagic acid glucuronoside, galloyl-bis-HHDP-glucose, genistin, hyperoside, epicatechin, procyanidin B1, procyanidin B2, dihydrokaempferol, gallocatechin, chlorogenic acid, genistein, ellagic acid glucuronoside, galloylquinic acid, gallic acid hexoside, and esculetin, that might contribute to the differences among the free and bound phenolic fractions of the three cultivars, serving as indicative markers distinguishing different cultivars to a certain extent.

We used Clustering correlation heatmap with signs to assess the relationship between the PCs and the bioactivity (Figures 4B, C). Previous studies have shown that ellagic acid and its derivatives, owing to the presence of many phenolic hydroxyl groups in their structures, positively impact the bioactivity (40, 41). The antioxidant activity of the free phenolic fraction positively correlated with the presence of 4-hydroxybenzoic acid, hyperoside, 4-hydroxycinnamic acid, isoflavones, flavonols, and their derivatives. Table 1 revealed significantly higher content of 4-hydroxybenzoic acid, hyperoside, 4-hydroxycinnamic acid, flavonols in “Tan xiang” than the other two cultivars, this may be the reason why it has the highest oxidative activity. Furthermore, paeonol showed is significantly associated with anti-inflammatory activity in FPs, which is important for neuroprotection (42). The potential anti-inflammatory activity of the bound phenolic fraction might be attributed to six PCs including quininic acid,4-hydroxycinnamic acid, galloylquinic acid, dihydrokaempferol, cinnamic acid and genistein, which was consistent with previous reports (43, 44). We observed that gallic acid, catechin, syringic acid, and nobiletin exhibit negative correlation in FPs and positive correlation in BPs of cytokine production, while corilagin and methyl ellagic acid pentoside exhibited opposite correlation. Combined with the results from Section 3.4, we concluded that, at the same dose, the anti-inflammatory capacity of BPs was superior to that of FPs. These findings suggested that other factors might impact the anti-inflammatory activities of FPs and BPs. On one hand, the bioactivities of the PCs might be influenced by their forms. For instance, galloyl-bis-HHDP-glucose and genistein only exist in free and bound forms, respectively. They have shown significant inhibition of IL-6 and NO production separately. On the other hand, the synergy among the different forms of PCs should not be neglected. For instance, galloylquinic acid and 4-hydroxycinnamic acid significantly inhibited the IL-6 production, but only in the bound form.

## 4 Conclusion

We identified 44 PCs in the three *Canarium album* L. cultivars and compared the free and bound phenolic levels among them. Among these cultivars, the “Tan xiang” cultivar harbored the highest levels of FPs. Moreover, the free phenolic fraction of this cultivar exhibited the highest antioxidant activity among all three cultivars. Furthermore, the TNF-α production of FPs was significantly decreased in “Xiang zhong” compared with “Na zhong”. In the assay of IL-6, production was significantly decreased in FPs of “Xiang zhong” compared with other cultivars. “Na zhong” and “Tan xiang” showed significant inhibitory ability in IL-6 production compared to “Xiang zhong” in BPs. We also explored the relationship between the PC composition of a cultivar and its contribution to the antioxidant and anti-inflammation activity. Of the identified 44 PCs, 30 PCs were found to contribute to the antioxidant activity of the cultivars. Furthermore, 8 PCs potentially contributed to the anti-inflammation activity in different form. In addition, we found that the bioactivities of the PCs might be influenced by their form or their synergy with other unknown PCs. Our findings highlighted the potential preventive and therapeutic effects of PCs and provided insights into optimizing the selection of Chinese olive cultivars with desired bioactivities. Future studies need to elucidate the mechanisms underlying the impact of polyphenols on the production and regulation of inflammatory factors. In conclusion, this study revealed the relevant differences in the phenolic compound profiles of different Chinese olive cultivars and identified the PCs that might contribute to the anti-inflammatory properties of these cultivars.

## Data availability statement

The original contributions presented in the study are included in the article/Supplementary material, further inquiries can be directed to the corresponding author.

## Author contributions

FH: Writing—original draft, Investigation, Methodology. YD: Formal analysis, Validation, Writing—review & editing. ZP: Data curation, Investigation, Writing—review & editing. HZ: Data curation, Software, Writing—review & editing. HL: Visualization, Writing—review & editing. JW: Resources, Software, Writing—review & editing. YS: Conceptualization, Writing— review & editing, Supervision. ML: Funding acquisition, Writing—review & editing, Conceptualization, Supervision.
