# Supplementary material for: The composition of phenolic compounds in Chinese olive (Canarium album L.) cultivars and their contribution to the anti-inflammatory properties of the cultivars
Source: Front Nutr. 2024 Feb 19;11:1334077. doi: 10.3389/fnut.2024.1334077 (PMC10910046; doi:10.3389/fnut.2024.1334077)
Supplement: Supplementary file 1 [file Data_Sheet_1.docx]

**Table of Supplementary material Ⅰ** Tentative identification of phenolic compounds(PCs) about the free phenolic(FPs) and bound phenolic(BPs) in *Canarium album* L.

| **ID** | **Tentative compound** | **RT**  **(min)** | **Molecular Formula** | **Ion mode** | **Ion peak**  **(*m*/*z*)** | **Fragment** | **Cultivars of *Canarium album* L.** | | | | | |
| --- | --- | --- | --- | --- | --- | --- | --- | --- | --- | --- | --- | --- |
|  |  |  |  |  |  |  | **‘Na zhong’** | | **‘Tan xiang’** | | **‘Xiang zhong’** | |
|  |  |  |  |  |  |  | **FP** | **BP** | **FP** | **BP** | **FP** | **BP** |
| **Hydroxybenzoic acids and derivatives** | | | | | | | | | | | | |
| 1 | Quinic acid | 1.67 | C_7_H_12_O_6_ | [M-H]^-^ | 191.05441 | 127.03812 | √ | √ | √ | √ | √ | √ |
| 2 | Galloylquinic acid | 2.66 | C_14_H_16_O_10_ | [M-H]^-^ | 343.06656 | 191.05504,  169.01312 | √ | √ | √ | √ | √ | √ |
| 3 | Gallic acid hexoside | 2.70 | C_13_H_16_O_10_ | [M-H]^-^ | 331.06583 | 169.01259,  124.01524 | √ | √ | √ | √ | √ | √ |
| 4 | Gallic acid | 3.91 | C_7_H_6_O_5_ | [M-H]^-^ | 169.01254 | 125.02260 | √ | √ | √ | √ | √ | √ |
| 5 | Vanillic acid hexoside | 4.01 | C_14_H_18_O_9_ | [M-H]^-^ | 329.08701 | 167.03391,  152.01031 | √ | - | √ | - | √ | - |
| 6 | Protocatechuic acid | 5.76 | C_7_H_6_O_4_ | [M-H]^-^ | 153.01767 | 109.02767 | √ | √ | √ | √ | √ | √ |
| 7 | Brevifolincarboxylic acid | 6.31 | C_13_H_8_O_8_ | [M-H]^-^ | 291.01473 | 247.02423 | √ | √ | √ | √ | √ | √ |
| 8 | 4-hydroxybenzoic acid | 6.41 | C_7_H_6_O_3_ | [M-H]^-^ | 137.02258 | 93.03278 | √ | √ | √ | √ | √ | √ |
| 9 | Syringic acid | 7.21 | C_9_H_10_O_5_ | [M-H]^-^ | 197.04469 | 153.05446 | √ | √ | √ | √ | √ | √ |
| **Hydroxycinnamic acids and derivatives** | | | | | | | | | | | | |
| 10 | Chlorogenic acid | 6.06 | C_16_H_18_O_9_ | [M-H]^-^ | 353.08887 | 191.05545 | √ | - | √ | - | √ | - |
| 11 | Caffeic acid | 6.81 | C_9_H_8_O_4_ | [M-H]^-^ | 179.0335 | 161.04378,  143.98611 | - | √ | - | √ | - | √ |
| 12 | Esculetin | 7.05 | C_9_H_6_O_4_ | [M-H]^-^ | 177.01831 | 177.01831,  148.96439 | - | √ | - | √ | - | - |
| 13 | 4-hydroxycinnamic acid | 8.78 | C_9_H_8_O_3_ | [M-H]^-^ | 163.03926 | 119.04892 | √ | √ | √ | √ | √ | √ |
| 14 | Ferulic acid | 9.53 | C_10_H_10_O_4_ | [M-H]^-^ | 193.04863 | 178.02556,  158.84500 | - | √ | - | √ | - | √ |
| 15 | Isoferulic acid | 10.21 | C_10_H_10_O_4_ | [M-H]^-^ | 193.81387 | 178.02557,  149.05905 | √ | √ | √ | √ | √ | √ |
| 16 | Cinnamic acid | 15.60 | C_9_H_8_O_2_ | [M+H]^+^ | 149.05971 | 131.92918,  103.05419 | √ | √ | √ | √ | √ | - |
| **Isoflavones and derivatives** | | | | | | | | | | | | |
| 17 | Genistin | 10.16 | C_21_H_20_O_10_ | [M-H]^-^ | 431.09824 | 269.04559 | √ | √ | √ | √ | √ | - |
| 18 | Genistein | 16.02 | C_15_H_10_O_5_ | [M-H]^-^ | 269.04559 | 197.05989  133.02831 | - | √ | - | √ | - | √ |
| **Flavones and derivatives** | | | | | | | | | | | | |
| 19 | Hyperoside | 9.12 | C_21_H_20_O_12_ | [M-H]^-^ | 463.08673 | 300.02603,  271.02298 | √ | √ | √ | √ | √ | √ |
| 20 | Astragalin | 10.12 | C_21_H_20_O_11_ | [M-H]^-^ | 447.09192 | 284.03146,  255.02794 | √ | - | √ | - | √ | - |
| 21 | Nobiletin | 16.85 | C_21_H_22_O_8_ | [M-H]^-^ | 401.08731 | 313.07007,  225.05339 | √ | √ | √ | √ | √ | √ |
| 22 | Amentoflavone | 17.06 | C_30_H_18_O_10_ | [M-H]^-^ | 537.08264 | 375.05087,  401.02982 | - | √ | - | √ | - | √ |
| **Flavonols and derivatives** | | | | | | | | | | | | |
| 23 | Isoquercitrin | 9.27 | C_21_H_20_O_12_ | [M-H]^-^ | 463.10280 | 300.02734,  271.02530 | √ | - | √ | - | √ | - |
| 24 | Kaempferol 3-O-glucoside | 10.00 | C_21_H_20_O_11_ | [M-H]^-^ | 447.09341 | 285.03931,  284.03247 | √ | - | √ | - | √ | - |
| 25 | Quercetin | 14.56 | C_15_H_10_O_7_ | [M-H]^-^ | 301.03381 | 273.93973,  178.99767 | √ | √ | √ | √ | √ | √ |
| 26 | Dihydrokaempferol | 15.73 | C_15_H_12_O_6_ | [M-H]^-^ | 287.22159 | 269.04532,  243.06570 | - | √ | - | √ | - | - |
| 27 | Kaempferol | 16.22 | C_15_H_10_O_6_ | [M-H]^-^ | 285.04047 | 257.04620,  151.00218 | - | - | √ | - | - | - |
| **Flavanols and derivatives** | | | | | | | | | | | | |
| 28 | Gallocatechin | 4.30 | C_15_H_14_O_7_ | [M-H]^-^ | 305.06528 | 167.03325 | √ | - | √ | - | √ | - |
| 29 | Procyanidin B1 | 5.44 | C_30_H_26_O_12_ | [M-H]^-^ | 577.13477 | 407.07788,  289.07257 | √ | - | √ | - | √ | - |
| 30 | Epigallocatechin | 5.55 | C_15_H_14_O_7_ | [M-H]^-^ | 305.06528 | 179.03383,  125.02285 | √ | - | √ | - | √ | - |
| 31 | Catechin | 5.88 | C_15_H_14_O_6_ | [M-H]^-^ | 289.07062 | 245.08060,  179.03340 | √ | √ | √ | √ | √ | √ |
| 32 | Procyanidin B2 | 6.57 | C_30_H_26_O_12_ | [M-H]^-^ | 577.13605 | 407.07721,  289.07196 | √ | - | √ | - | √ | - |
| 33 | Epicatechin | 6.95 | C_15_H_14_O_6_ | [M-H]^-^ | 289.07050 | 245.08046 | √ | - | √ | - | √ | - |
| **Other** **polyphenols and derivatives** | | | | | | | | | | | | |
| 34 | Geraniin isomers | 6.71 | C_41_H_28_O_27_ | [M-H]^-^ | 951.07397 | 765.05890,  300.99860 | √ | √ | √ | √ | √ | √ |
| 35 | Corilagin (1-O-galloyl-3,6-(R)-HHDP-β-d-glucose) | 6.79 | C_9_H_10_O_4_ | [M-H]^-^ | 633.07343 | 300.99860  275.01981 | √ | √ | √ | √ | √ | √ |
| 36 | Galloyl-bis-HHDP-glucose | 6.86 | C_41_H_28_O_26_ | [M-H]^-^ | 935.07928 | 633.07623,  463.05222,  300.99893 | √ | - | √ | - | √ | - |
| 37 | Geraniin | 7.78 | C_41_H_28_O_27_ | [M-H]^-^ | 951.07397 | 765.05890,  300.99860 | √ | √ | √ | √ | √ | √ |
| 38 | Ellagic acid pentoside | 7.85 | C_19_H_14_O_12_ | [M-H]^-^ | 433.04089 | 300.99850 | √ | √ | √ | √ | √ | √ |
| 39 | Ellagic acid glucuronoside | 8.37 | C_20_H_14_O_14_ | [M-H]^-^ | 477.10364 | 433.11401,  315.01450,  300.99847 | √ | - | √ | - | √ | - |
| 40 | Methyl-ellagic acid glucuronoside | 8.67 | C_21_H_16_O_14_ | [M-H]^-^ | 491.04654 | 315.01434,  300.99850 | √ | - | √ | - | √ | - |
| 41 | Ellagic acid | 8.84 | C_14_H_6_O_8_ | [M-H]^-^ | 300.99756 | 257.00873 | √ | √ | √ | √ | √ | √ |
| 42 | Methyl ellagic acid pentoside | 9.83 | C_20_H_16_O_12_ | [M-H]^-^ | 447.05676 | 315.01450,  300.99802 | √ | √ | √ | √ | √ | √ |
| 43 | Syringaldehyde | 10.75 | C_9_H_10_O_4_ | [M-H]^-^ | 181.97128 | 136.90796 | √ | √ | √ | √ | √ | √ |
| 44 | Paeonol | 17.28 | C_9_H_10_O_3_ | [M+H]^+^ | 167.10587 | 149.09552,  121.10091 | √ | √ | √ | √ | √ | √ |

RT, retention time; Ion peak, product ions were generated by the fragmentation of ion mode([M-H]^-^ or [M+H]^+^) ; √, identified; -, not found.

**Figure of Supplementary material Ⅰ** Total ion chromatograms (TICs) of phenolic extracts by UPL C-Q-Exactive Orbitrap/MS analysis.


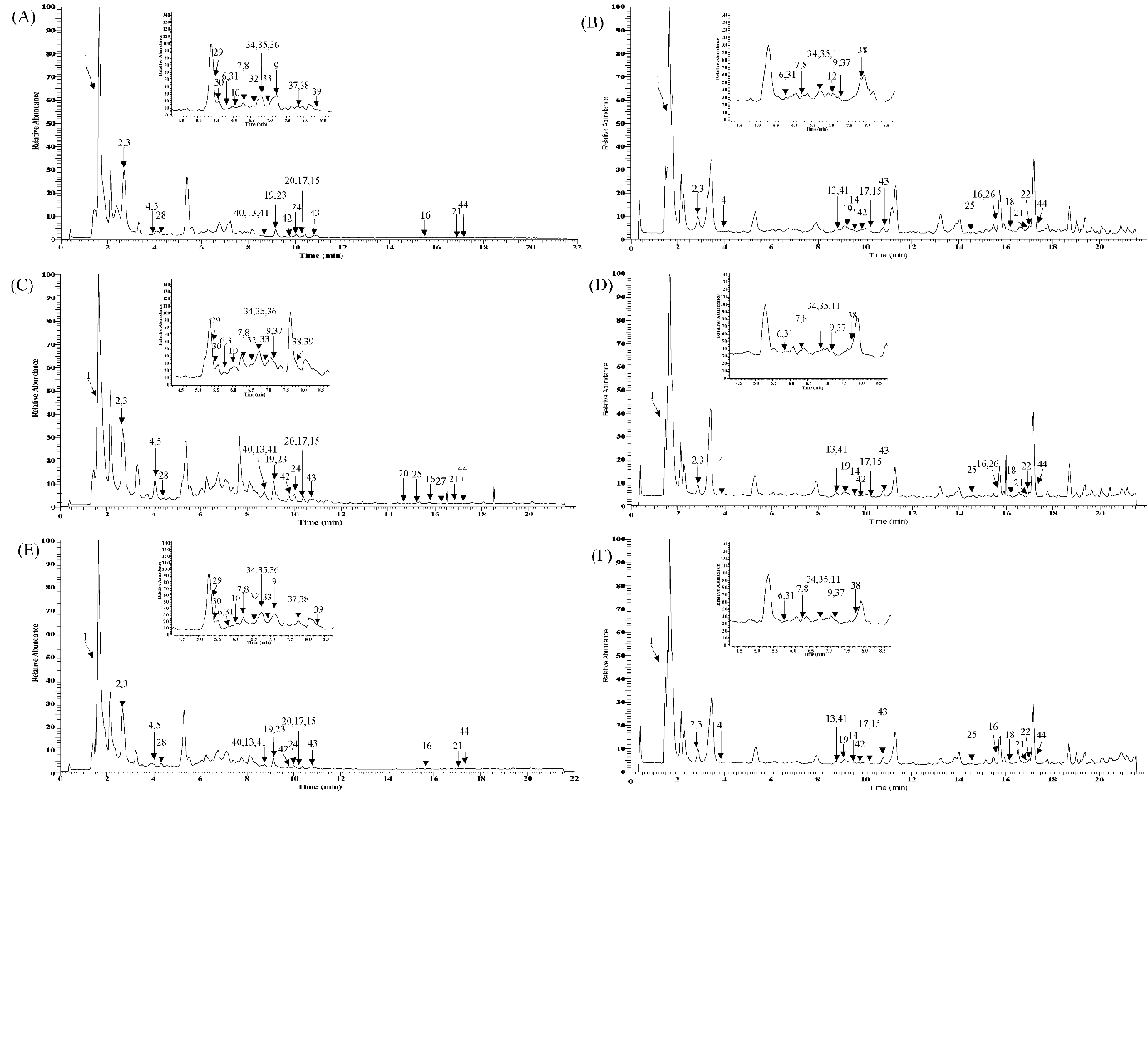
(A) Free phenolics of “Na zhong” (B) Bound phenolics of “Na zhong” (C) Free phenolics of “Tan xiang” (D) Bound phenolics of “Tan xiang” (E) Free phenolics of “Xiang zhong” (F) Bound phenolics of “Xiang zhong”. Numbers represented compounds corresponding Table of Supplementary material Ⅰ.

**Table of Supplementary material** **Ⅱ** **Calibration curves used for UPLC-MS/MS quantification of polyphenols.**

|  | **Phenolic compounds** | **Calibration curves** | **Correlation coefficients (r^2^)** | **Linear ranges (ng/mL)** |
| --- | --- | --- | --- | --- |
| 1 | Quinic acid | Y = 474066+37436.7*X | 0.9999 | 34.704-4993.240 |
| 2 | Gallic acid | Y = -511402+43335.8*X | 1.0000 | 12.121-5002.742 |
| 3 | Vanillic acid | Y = -144667+2949.35*X | 0.9993 | 88.094-5034.729 |
| 4 | Syringic acid | Y = -499429+6540*X | 0.9984 | 83.435-5028.414 |
| 5 | Protocatechuic acid | Y = 14594.8+32044.2*X | 0.9999 | 0.940-4999.407 |
| 6 | 4-Hydroxybenzoic acid | Y = 1555500+38341.5*X | 0.9990 | 13.365-4974.789 |
| 7 | Chlorogenic acid | Y = 221223+20077.3*X | 0.9996 | 31.167-4986.573 |
| 8 | Esculetin | Y = 10420200+100125*X | 0.9957 | 217.381-4947.619 |
| 9 | Caffeic acid | Y = 1055950+78625.2*X | 0.9998 | 37.295-4992.554 |
| 10 | 4-Hydroxycinnamic acid | Y = 1713660+71899.8*X | 0.9998 | 1.331-4991.344 |
| 11 | Ferulic acid | Y = 148895+26664*X | 1.0000 | 6.387-4998.020 |
| 12 | Isoferulic acid | Y = -61412.9+2946*X | 0.9997 | 23.991-5011.701 |
| 13 | Coumaric acid | Y = -57752.9+1416.35*X | 0.9998 | 70.077-5020.944 |
| 14 | Genistin | Y = 20947.4+3102.9*X | 1.0000 | 2.736-4996.989 |
| 15 | Genistein | Y = 13657100+170781*X | 0.9976 | 223.941-4961.438 |
| 16 | Hyperoside | Y = 546245+18544.5*X | 0.9996 | 23.182-4986.088 |
| 17 | Astragalin | Y = 1404690+31513.4*X | 0.9990 | 8.536-4977.245 |
| 18 | Isoquercitrin | Y = 768870+22520.7*X | 0.9993 | 17.932-4984.228 |
| 19 | Quercetin | Y = 6051820+55401.9*X | 0.9938 | 183.477-4936.325 |
| 20 | Kaempferol | Y = 10181600+104115*X | 0.9961 | 225.559-4950.054 |
| 21 | (-)-Gallocatechin | Y = -221452+19467.3*X | 0.9998 | 14.548-4997.684 |
| 22 | Procyanidin B1 | Y = -42719.6+6752.17*X | 1.0000 | 10.199-5000.002 |
| 23 | Catechin | Y = -252455+32115.4*X | 0.9999 | 14.309-5002.795 |
| 24 | Procyanidin B2 | Y = -244115+8836.59*X | 0.9999 | 31.771-5007.390 |
| 25 | Epicatechin | Y = 1115040+40844.7*X | 0.9996 | 23.416-4983.471 |
| 26 | Corilagin | Y = -6482770+10831.7*X | 0.9993 | 616.245-50134.213 |
| 27 | Paeonol | Y = 86602.9+1532.31*X | 0.9999 | 60.713-4997.408 |
| 28 | Ellagic acid | Y = -1512580+19868.7*X | 0.9982 | 78.744-5016.388 |
| 29 | Syringaldehyde | Y = -317083+6364.65*X | 0.9992 | 57.977-5020.643 |
